# Agranulocytosis secondary to zinc excess: Clinically relevant observations, including response to G‐CSF and oral copper

**DOI:** 10.1002/ccr3.3301

**Published:** 2020-09-10

**Authors:** Paul L. Weiden, Marlowe Dunker, David J. Corwin

**Affiliations:** ^1^ Bartlett Regional Hospital Juneau Alaska; ^2^ CellNetix Pathology Seattle Washington

**Keywords:** acute medicine, haematology, toxicology

## Abstract

Anemia and leukopenia because of copper deficiency can be mistaken for myelodysplasia. Key issues, including response to G‐CSF and oral copper, are discussed. This case illustrates a significant deleterious effect of excessive zinc consumption.

## Graphical abstract

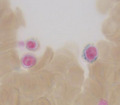

## 1 INTRODUCTION

The lay press and the internet extol the benefits of zinc supplementation: “When cold and flu season hits, it's wise to have an arsenal … on hand to fortify your immune system….Zinc benefits your immune system.”^1^ “…12 percent of people in the US…[and] of those 65 or older, closer to 40%, do not consume enough zinc.”^2^ “Consequently, oral zinc supplementation demonstrates the potential to improve immunity…”^3^ Less well publicized are the potential risks and untoward consequences of excessive zinc intake.^4^, ^5^, ^6^, ^7^

## 2 CASE REPORT

**Figure 1:**
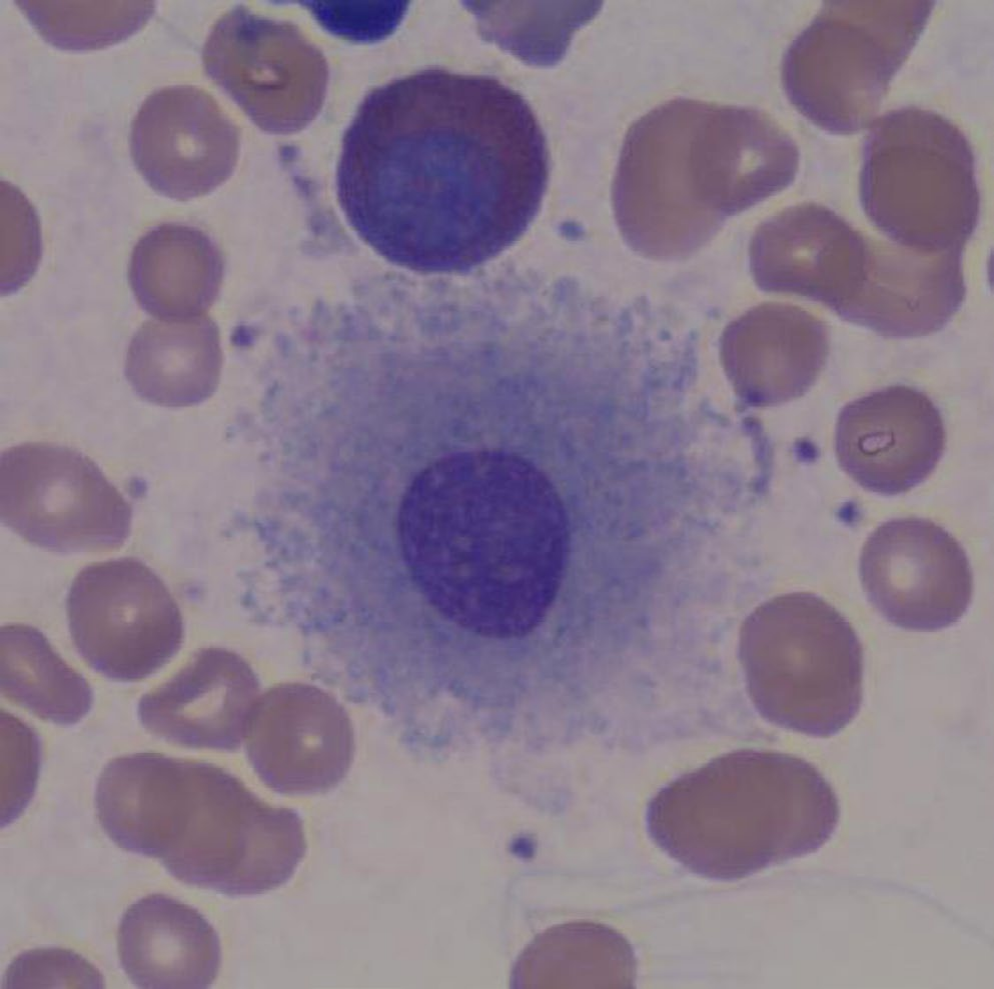
An abnormal, uninucleate megakaryocyte. This dysplastic change was noted even though the peripheral platelet count was normal. (Wright‐Giemsa stain)

**Figure 2:**
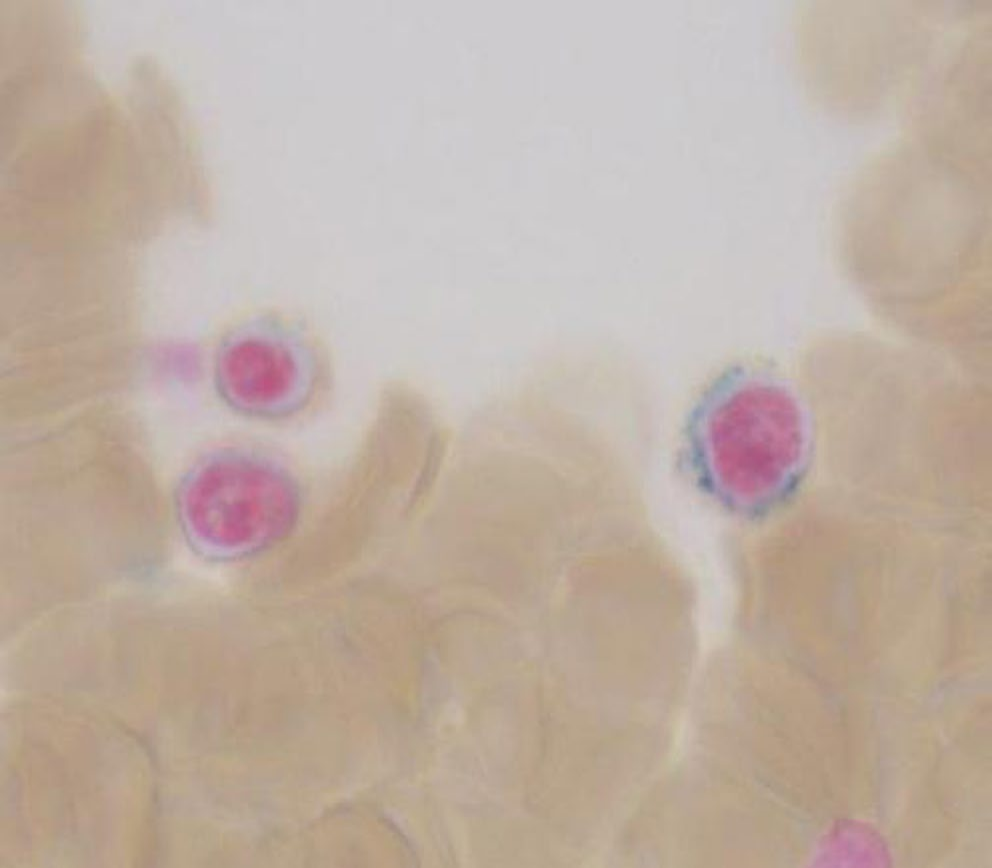
Three sideroblasts with dysmorphic iron granules (shown in blue) surrounding the nucleus of maturing red blood cells (normoblasts). (Prussian blue stain)

A 69‐year‐old male was evaluated for weakness, dyspnea on exertion, and chest pain. CBC showed a hematocrit of 22%, mean corpuscular volume (MCV) of 97 fL, white cell count of 1000/µL, absolute neutrophil count (ANC) of 200/µL, and platelet count of 185,000/µL (see Table 1). The patient was transfused 2 units of red blood cells with symptomatic improvement. Over the next 3 days, his ANC declined and he was referred with the presumptive diagnosis of a myelodysplastic syndrome (MDS). Medical history, prescribed medications, and physical examination were unremarkable; alcohol intake was minimal. His ANC had declined further to 0/µl while his platelet count remained normal (185 000/µL). Marrow aspirate showed left‐shifted and dysmorphic erythroid proliferation, left‐shifted myeloid proliferation with maturation arrest, and abnormal megakaryocytes (Figure 1). Most striking was the presence of dysmorphic iron incorporation in 51% of the normoblasts, that is, partial or complete “ring sideroblasts” (Figure 2). Flow cytometry showed a mildly increased proportion of myeloblasts comprising 5.3% of total leukocytes. Cytogenetics were normal. The pathologist favored MDS but also noted that “given the ring sideroblasts and vacuolar changes seen among red cell precursors…the possibility of drug toxicity should be considered and a serum copper level obtained.”

**Table 1:** Time Line of Patient's Course Related to Day 1, Presentation In ER

Day	Clinical events	Copper replacement	Hematocrit, %	Absolute neutrophils /µL	Serum Co, µg/dL (Normal = 72‐166)	Serum Zn, µg/dL (Normal = 56‐134)
‐123	Routine CBC		43.0	4700	‐	‐
1	Weakness, dyspnea, chest pain		22.3	200	‐	‐
2	After 2 units RBC		27.4		‐	‐
4			28.0	100	‐	‐
6	Marrow aspirate First dose, G‐CSF^a^ Patient discontinued supplements		25.0	0	‐	‐
7	2 units RBC, G‐CSF				‐	‐
10	G‐CSF		32.4	400	‐	‐
12	G‐CSF		31.6	2600	0	266
14	Last G‐CSF				‐	‐
17		Start po copper^b^, 8mg/day	31.4	1800	‐	‐
21		Start IV copper^c^	31.3	2100	31	236
24		IV copper	32.9	2800	‐	‐
26		IV copper				
28		Last IV copper Decrease po copper to 4mg/day	36.0	3400	79	256
48		Decrease po copper to 2mg/day	40.8	5400	86	149
62		Discontinue oral copper	42.6	5000	150	174
117			49.3	3900	97	85

^a^ 480 µg tbo‐filgrastim, subcutaneously.

^b^ Copper bisglycinate (Thorne Research).

^c^ Serum copper level obtained prior to IV infusion of cupric chloride, 2.4 mg in normal saline.

A CBC obtained 4 months earlier was normal with a hematocrit of 43% and a neutrophil count of 4700/µL. The patient also reported that he had been taking zinc supplements, 16‐20 tablets/day that were discontinued on the day of his marrow aspirate. As shown in Table 1, initial copper level was 0 µg/dL (normal = 72‐166 µg/dL), zinc level 266 µg/dL (normal = 56‐134 µg/dL), and ceruloplasmin was <3.0 µg/dL (normal 16‐31 µg/dL).

The patient was a retired business executive previously based in a major European capital. He had traveled widely internationally including to regions with poor hygiene and unsanitary food practices. He began taking zinc to “strengthen his immune system” 14 years previously, initially taking zinc gluconate 250 mg/d, then 500 mg/d and then 800‐1000 mg (8‐10 tablets)/d. The tablets were labeled as “zinc, 100 mg, as zinc gluconate.” (The recommended daily dietary allowance for zinc in the United States is 8 mg for women and 11 mg for men.^8^) Four months prior to presentation, he switched to tablets purchased on the internet: “zinc, 50 mg (from zinc sulfate heptahydrate 220 mg)” and took 16‐20 tablets/day.

The patient's profound neutropenia responded rapidly to G‐CSF (Table 1). His serum copper level responded rapidly to oral copper supplementation, increasing from 0 to 31 µg/µL in 4 days, even though his serum zinc level remained above normal. He was given four doses of copper intravenously, which was discontinued when the results of his serum copper were available and his ANC had returned to normal. The dose of oral copper was slowly decreased and discontinued when his serum copper was normal (Table 1). Two months later, on no medications or supplements, his CBC, copper, and zinc were normal.

## 3 DISCUSSION

Although acquired copper deficiency, either as a result of nutritional deficiency, most often related to gastric bypass surgery,^10^, ^11^ or excessive zinc supplementation^4^, ^5^, ^6^, ^7^ is well described in the medical literature, this diagnosis can easily be missed in clinical practice. In fact, patients presenting with neutropenia and anemia secondary to copper deficiency have been misdiagnosed as having MDS and been referred for stem cell transplantation.^7^ In the patient reported here, profound neutropenia with only moderate anemia, a normal MCV, a normal platelet count, and an entirely normal CBC 4 months previously seemed inconsistent with the diagnosis of MDS. The abnormalities seen on the marrow aspirate, while consistent with MDS, raised the possibility of copper deficiency, confirmed by the subsequent serum copper level of zero. Additional history then identified the etiology of the copper deficiency, that is, excess zinc supplementation. The mechanism by which excess zinc intake leads to copper deficiency is incompletely understood, but is thought to result from induction of excess synthesis of the intracellular ligand metallothionein (MTO) in enterocytes.^9^ The excess MTO then binds copper, resulting in both less absorption into the plasma and excess excretion in the feces.^5^, ^8^, ^9^ The mechanisms by which plasma copper deficiency results in its characteristic hematologic abnormalities (anemia and leukopenia without thrombocytopenia) are even less well understood, but are thought to be related to decreased activity of copper‐dependent enzymes.^7^, ^10^, ^11^

Several unique observations in the course of this patient are particularly worthy of attention:

1. Although significant neutropenia related to copper deficiency and/or excess zinc has been reported before, agranulocytosis (total absence of neutrophils in the peripheral blood) has not been previously reported.
2. Treatment with G‐CSF has also not been previously reported (and is not necessarily indicated when neutropenia is less profound). Nevertheless, the rapid response of the profound neutropenia to G‐CSF, while perhaps not surprising given the abundance of myeloid precursors in the marrow, is an important clinical observation.
3. Both oral and IV copper supplementation have been used previously in treatment of similar patients, but the rapid response to oral copper in this patient before administration of IV copper suggests that oral copper, more readily available as a nutritional supplement and less expensive that IV copper, may be sufficient therapy, at least in individuals with a normal intestinal tract.
4. The data in this patient (Table 1) clearly indicate that a high serum level of zinc does not impede the reversal of copper deficiency by oral administration of copper, confirming previous reports that although the decrease in serum copper levels results primarily from impairment of copper absorption,^5^, ^6^, ^10^ the impairment can be overcome by supplemental oral copper. This observation is in contrast to a previous report^12^ which may account for the frequent administration of IV copper in patients with zinc‐induced copper deficiency.
5. High levels of serum zinc persisted in this patient for more than two months without obvious clinical or laboratory consequences.
6. Finally, “zinc supplementation” does not address the many forms of zinc that are available “over the counter,” including zinc acetate, sulfate, picolinate, gluconate, glycinate, orotate, and citrate.^1^ Clearly, this patient tolerated large amounts of zinc gluconate for many years without hematological effect, but zinc sulfate relatively rapidly resulted in marked hematologic abnormalities.

While beyond the scope of this discussion, the safety of readily available supplements including zinc, especially if taken in more that the recommended dosage, has significant individual and public health consequences.^13^

## CONFLICT OF INTEREST

None declared.

## AUTHOR CONTRIBUTIONS

PW: coordinated the care of the patient, conceived of and wrote the paper. MD: assisted in the care of the patient and collection of clinical data. DC: made the original critical observation of the marrow pathology, assisted in eliminating other contributing causes of the hematological abnormalities, and reviewed the manuscript.

## ETHICAL APPROVAL

None required; the patient did consent to the publication of this Case Report.
